# Breast cancer clinical trials in low- and middle-income countries (LMICs)

**DOI:** 10.3332/ecancer.2026.2112

**Published:** 2026-04-14

**Authors:** Adnan-Mustafiz Chowdhury, Sattam A Halaseh, Debora Joseph, Abel I Abel, Nikita Jha, Amro Al-Karadsheh, Hakam Al-Karadsheh, Omar A Salem, Sama F Salaitah, Noor M Arman, Georges Rizkallah

**Affiliations:** 1 General Surgery Department, Dorset County Hospital NHS Foundation Trust, Williams Avenue, Dorchester, Dorset DT1 2JY, UK; 2 University Hospitals Sussex NHS Foundation Trust, Worthing Hospital, Worthing, West Sussex BN11 2DH, UK; 3 United Lincolnshire Hospitals NHS Trust, Lincoln County Hospital, Lincoln, Lincolnshire LN2 4AX, UK; 4 East Sussex Healthcare NHS Trust, Conquest Hospital, St Leonards-on-Sea, East Sussex TN37 7PT, UK; 5 The Hashemite University, Damascus Hwy, Zarqa 13133, Jordan; 6 University of Jordan, Amman 11942, Jordan; 7 Salisbury District Hospital, Salisbury, Wiltshire, Salisbury SP2 8BJ, UK

**Keywords:** breast cancer, clinical trials, low- and middle-income countries, global health disparities, pharmaceutical funding

## Abstract

**Background:**

Breast cancer is the most diagnosed malignancy worldwide and a leading cause of cancer-related mortality in women. Clinical trials underpin advances in prevention, diagnosis and treatment; however, the landscape of breast cancer trials in low- and middle-income countries (LMICs) remains insufficiently characterised. This study aimed to describe the scope, design, interventions and funding of breast cancer clinical trials conducted in LMICs between January 2010 and December 2020.

**Methods:**

We searched the Cochrane Central Register of Controlled Trials on 15 November 2023 for trials using the keywords ‘breast cancer’, ‘breast adenocarcinoma’ and ‘breast tumour’. Trials were classified by World Bank income category. Extracted variables included study design, primary purpose, time perspective, observational model, trial phase, intervention type, sample size, status, reason for termination, funding source and centre type. Descriptive statistics summarised trial characteristics.

**Results:**

A total of 333 breast cancer trials were identified; Two trials (0.6%) were conducted in low-income countries and 331 (99.4%) in lower-middle-income countries; no trials conducted exclusively in upper-middle-income countries were identified within the registry during the study period. Most trials were interventional (85.3%), prospective (95.8%) and treatment-focused (71.5%). Drug-based interventions predominated (64.9%). Funding was primarily from pharmaceutical or biotechnology companies (53.2%), followed by academic or institutional (university-based) sources (39.6%). Only 7.5% of trials addressed prevention, screening and supportive care. Multicentre trials accounted for 53.8%, single-centre 46.3%.

**Conclusion:**

Breast cancer trials in LMICs are predominantly interventional, pharmaceutically-funded and concentrated in lower-middle-income settings. There remains a substantial paucity of trials from low-income countries and in non-treatment domains such as prevention, screening and supportive care.

## Background

Breast cancer is the most commonly diagnosed malignancy worldwide, with an estimated 2.3 million new cases and 670 000 deaths reported in 2020, accounting for nearly one quarter of all cancer diagnoses in women and 16% of cancer-related mortality [1]. While high-income countries (HICs) have witnessed substantial improvements in early detection, systemic therapies and survivorship care, these gains have not been uniformly realised in low- and middle-income countries (LMICs) [2, 3]. In many LMIC settings, patients present with more advanced disease, access to standard-of-care diagnostics and treatments is limited and health systems face significant infrastructural and financial constraints [4]. According to data from GLOBOCAN, although breast cancer incidence is highest in HICs, such as those in North America and Europe, mortality rates are disproportionately elevated in LMICs, particularly in sub-Saharan Africa and parts of South America [4]. This disparity is not only due to differences in risk factors like lifestyle and genetics but also reflects systemic barriers to early detection and effective treatment. Consequently, incidence-to-mortality ratios are significantly lower in LMICs (e.g., between 1.77 and 1.94 in sub-Saharan Africa) compared to 6.39 in Northern Europe and 6.73 in North America [5].

Clinical trials are essential for generating high-quality evidence to inform prevention, diagnosis and treatment strategies in breast cancer care. However, the trial landscape in LMICs remains poorly characterised. Despite the growing disease burden, the number and variety of clinical trials conducted in these regions are limited. Most trials are concentrated in a few upper-middle-income countries and are often funded or led by institutions based in HICs [6].

Mapping the current clinical trial landscape is essential for identifying context-specific research gaps and informing more equitable global health strategies. Understanding differences in clinical characteristics, such as age at diagnosis, stage of presentation and tumour subtypes, is crucial for improving outcomes in LMICs. Additionally, the focus of ongoing trials, whether on treatment, screening, prevention or supportive care must better reflect local health priorities and system capacities.

Currently, most breast cancer trials in LMICs focus on pharmacological treatments, with limited attention to screening, radiotherapy or supportive care [7]. Research efforts are often hindered by systemic challenges, including inadequate infrastructure, limited access to radiotherapy and constrained funding. Moreover, countries with the highest disease burden frequently host few trials, highlighting a disconnect between research activity and local health needs. Without intervention, LMICs may face a crisis point where the rising cost of treatment and increasing incidence collide with limited access and affordability. To avoid this, the development of affordable, simple and accessible alternatives is needed. Importantly, advancing global equity in breast cancer outcomes will require more robust, evidence-based research that specifically addresses the unique circumstances of LMICs [7].

This study aims to characterise the scope, design, focus of interventions and funding sources of breast cancer clinical trials in LMICs over a ten-year period, providing a foundation for more inclusive and context-appropriate research efforts.

## Methods

### Search strategy and selection criteria

We performed a registry-based analysis using the Cochrane Central Register of Controlled Trials (CENTRAL) on 15 November 2023. CENTRAL aggregates trial records from multiple sources, including ClinicalTrials.gov and several national and regional registries. WHO International Clinical Trials Registry Platform (ICTRP) was not searched independently.

Search terms included ‘breast cancer’, ‘breast adenocarcinoma’ and ‘breast tumour’. Eligible studies were clinical trials registered between January 2010 and December 2020 that enrolled adult participants with breast cancer and conducted in one or more LMICs, as defined by the World Bank Atlas (July 2023 classification [8]).

Phase 0 and pharmacokinetic-only studies, trials conducted exclusively in HICs and records lacking sufficient methodological information were excluded.

### Data extraction and quality control

Data were extracted using a standardised proforma. Variables included country and income classification, study design (interventional or observational), primary purpose, time perspective, intervention type, trial phase, sample size, funding source and centre type. Retrospective studies were defined as those in which data had been collected prior to trial registration and subsequently analysed without prospective follow-up.

Trial completion status and dates were extracted as reported in registry entries at the time of data collection; subsequent updates were not captured.

### Statistical analysis

Descriptive statistics were used to summarise trial characteristics. Categorical variables are reported as frequencies and percentages, and continuous variables as medians with interquartile ranges.

## Results

### Trial distribution

A total of 333 breast cancer clinical trials conducted in LMICs were identified. Two trials (0.6%) were conducted in low-income countries and 331 (99.4%) in LMICs (Table 1); No trials conducted exclusively in upper-middle-income countries were identified within the registry during the study period, which may reflect registry limitations or income classification at the time of trial registration.

When grouped by WHO region, most trials were conducted in the Eastern Mediterranean (38.1%) and South-East Asia (27.6%) regions, with relatively few studies from Africa (4.5%) and the Americas (1.8%) (Table 2).

### Study design and objectives

Most trials were interventional (85.3%), with observational studies accounting for 14.7% (Table 3). Treatment was the primary objective in 71.5% of trials, followed by supportive care (6.6%), prevention (6.0%) and screening (1.5%). The majority employed a prospective design (95.8%), with retrospective (1.5%) and cross-sectional (1.8%) designs less common. Among observational studies, cohort designs (44%) were the most common, followed by case-control (13.6%) and other models (Table 4).

### Intervention types

Drug-based interventions predominated, accounting for 64.9% of trials. Other interventions included radiotherapy (4.8%), combined treatment modalities (3.0%), procedures, diagnostic tests, behavioural interventions and devices (Table 5).

Among drug-focused trials (*n* = 216), 176 (81.4%) evaluated systemic anticancer therapies, including cytotoxic chemotherapy, endocrine agents and targeted treatments, either alone or in combination.

### Sample size

Over half of trials (54.1%) planned to recruit fewer than 500 participants (<100 in 13.0%, 101–500 in 41.1%) (Table 6).

### Funding, collaboration and completion status

Funding was primarily provided by pharmaceutical or biotechnology companies (53.2%), followed by academic or institutional (university-based) sources (39.6%) (Table 6). Other funding sources accounted for 6.6%.

Multicentre trials comprised 53.8% of studies (Table 7). Where collaboration data were available (*n* = 176), most multicentre trials involved partnerships spanning LMICs, UMICs and HICs (77.7%), while exclusively LMIC-based collaborations were uncommon (6.3%).

## Discussion

This analysis demonstrates that breast cancer clinical trial activity in LMICs between 2010 and 2020 was heavily concentrated in lower-middle-income countries (99.4%), with minimal representation from low-income settings (0.6%). Across the study period, trials were predominantly interventional (85.3%), prospective (95.8%) and randomised (72.5%), and largely focused on drug-based therapeutic interventions (64.9%).

The dominance of systemic therapies, including chemotherapy backbones, endocrine agents and targeted treatments, reflects a research agenda driven largely by pharmaceutical and biotechnology funding, which accounted for over half of all trials. In contrast, prevention, screening and supportive care were markedly under-represented, together comprising fewer than one-fifth of studies. Supportive care research, in particular, was sparse and largely observational, despite its potential to improve quality of life, treatment adherence and outcomes in resource-constrained settings.

These findings mirror observations from other malignancies in LMICs, including prostate cancer, where clinical trial activity is similarly skewed towards industry-sponsored, treatment-oriented studies conducted predominantly in middle-income countries [9]. Given that breast cancer carries a substantial burden in many LMICs – often presenting at more advanced stages and with distinct biological subtypes – the paucity of prevention, early detection and supportive-care research is particularly concerning. Evidence generated in HICs may not generalise to these contexts owing to differences in resource availability, treatment access, tumour genomics and patient comorbidities.

Several structural barriers likely contribute to these patterns. The predominance of pharmaceutical funding highlights the reliance on commercial interests to drive research in LMICs, which may bias trial design towards novel therapeutics rather than public-health interventions. The high costs of oncology trials frequently exceed the annual per-capita gross national income in many LMICs, constituting a formidable barrier to locally led research [10]. Moreover, regulatory complexity, limited research infrastructure and competing clinical duties further impede trial initiation and completion in resource-constrained settings. In addition, challenges in participant recruitment – including limited public awareness of clinical research and socioeconomic barriers to enrolment – may further hinder trial initiation and completion in these settings [11].

To address these gaps, a multifaceted approach is essential. International and local stakeholders must invest in research infrastructure, streamline regulatory pathways and foster equitable partnerships that prioritise capacity-building and local leadership. Diversification of funding – through governmental and philanthropic channels – would support studies of screening, prevention and supportive care. Strengthening training in research methodology and trial management for clinicians and trainees in LMICs is also critical. Finally, journals and professional societies should continue efforts to reduce publication barriers and promote the visibility of high-quality research from under-represented regions.

By broadening the scope of breast cancer clinical trials in LMICs and ensuring that research agendas align with local needs, the global oncology community can generate the context-relevant evidence necessary to improve outcomes and reduce disparities worldwide.

## Limitations

This study has several limitations. Reliance on CENTRAL without independent searching of WHO ICTRP or regional trial registries may have resulted in missed studies, particularly from upper-middle-income countries. Registry data did not permit systematic assessment of publication outcomes, outcome direction or the depth of international collaboration. Finally, trial characteristics were analysed as reported at the time of data extraction and may have changed subsequently.

## Conclusion

Breast cancer clinical trials in LMICs are predominantly interventional, pharmaceutically funded, concentrated in lower-middle-income countries, with notable gaps in low-income settings and non-treatment research domains. Strengthening research capacity, diversifying funding sources and fostering equipments.

International collaborations are essential to align future trials with local health priorities and reduce global disparities in breast cancer outcomes.

## Author contributions

Adnan-Mustafiz Chowdhury: Conceptualisation, Methodology, Formal Analysis, Writing – Original Draft Preparation, Writing – Original Draft Preparation, Writing – Review & Editing, Project Administration

Sattam A. Halaseh: Conceptualisation, Methodology, Formal Analysis, Writing – Original Draft Preparation, Writing – Original Draft Preparation, Writing – Review & Editing, Project Administration

Debora Joseph: Literature Review, Writing – Review & Editing

Abel I. Abel: Writing – Review & Editing

Nikita Jha: Writing – Review & Editing

Amro Al-karadsheh: Data Collection, Writing – Review & Editing

Hakam Al-karadsheh: Data Collection, Writing – Review & Editing

Omar A. Salem: Data Collection, Writing – Review & Editing

Noor M. Arman: Data Collection, Writing – Review & Editing

Georges Rizkallah: Data Collection, Writing – Review & Editing

## Conflicts of interest

The authors declare no competing interests.

## Funding

No external funding was received.

## Figures and Tables

**Table 1.** Trial distribution by income category. Number and proportion of breast cancer clinical trials conducted in low-income and lower-middle-income countries between 2010 and 2020.

Income category	n	%
Low-income countries	2	0.6
Lower-middle-income countries	331	99.4

**Table 2.** Trial distribution by WHO region. Number and proportion of breast cancer clinical trials conducted in low- and middle-income countries between 2010 and 2020, stratified by World Health Organisation (WHO) region.

WHO region	n	%
EMRO	127	38.1%
SEARO	92	27.6%
EURO	63	18.9%
WPRO	30	9.0%
AFRO	15	4.5%
AMRO	6	1.8%

**Table 3.** Study design and objectives. Characteristics of included trials according to study type, primary objective and time perspective.

Characteristic	n	%
Study type		
Interventional	284	85.3
Observational	49	14.7
Primary purpose		
Treatment	238	71.5
Supportive care	22	6.6
Prevention	20	6.0
Screening	5	1.5
Time perspective		
Prospective	319	95.8
Retrospective	5	1.5
Cross-sectional	6	1.8

**Table 4.** Observational models. Distribution of observational study designs among included breast cancer clinical trials.

Model	n	%
Cohort	44	44.0
Case-control	13	13.6
Case-crossover	1	2.3
Case-only	8	8.2
Other	18	18.2

**Table 5.** Intervention types. Distribution of breast cancer clinical trials in low- and middle-income countries by type of intervention.

Type	n	%
Drug	216	64.9
Radiotherapy	16	4.8
Combined modalities	10	3.0
Device	4	1.2
Diagnostic test	4	1.2
Behavioural	3	0.9
Dietary supplement	4	1.2
Procedure	5	1.5
Exercise	1	0.3
Virtual reality-based	1	0.3

**Table 6.** Sample size and funding. Distribution of included trials by planned sample size and reported funding source.

Category	n	%
Sample size		
<100	43	13.0
101–500	137	41.1
501–1,000	48	14.4
>1,001	44	13.2
Funding source		
Pharma/Biotech	177	53.2
Institutional	132	39.6
Other (personal etc.)	22	6.6

**Table 7.** Centre type. Distribution of included trials by centre type, comparing single-centre and multicentre studies.

Centre type	n	%
Multicentre	179	53.8
Single centre	154	46.3
